# Glycolysis-Driven Immune Subtypes in the Tumor Microenvironment Determine Clinical Outcomes in Diffuse Large B-Cell Lymphoma

**DOI:** 10.3390/cancers18010075

**Published:** 2025-12-25

**Authors:** Ying Du, Zhenyu Liu, Chuanyan Liu, Yining Yuan, Mengkun Fang, Tong Zhou, Mengge Pan, Weilu Xu, Xu Liu, Peipei Xu

**Affiliations:** 1 Department of Hematology, Nanjing Drum Tower Hospital, Affiliated Hospital of Medical School, Nanjing University, Nanjing 210008, China; 18761868919@163.com (Y.D.);; 2 Department of Hematology, Nanjing First Hospital, Nanjing Medical University, Nanjing 210006, China; 3 General Medicine Department, Nanjing Drum Tower Hospital, Affiliated Hospital of Medical School, Nanjing University, Nanjing 210008, China

**Keywords:** diffuse large B-cell lymphoma, glycolysis modification, immune cell infiltration, cluster analysis

## Abstract

**Background:** Diffuse large B-cell lymphoma (DLBCL), the most common B-cell non-Hodgkin lymphoma, remains challenging because more than one-third of patients fail to achieve durable remission with frontline therapy; therefore, a systematic and comprehensive understanding of its underlying biology is essential to improve risk stratification and treatment outcomes. **Methods:** Based on 30 glycolysis-related genes selected from 72 candidates in the MSigDB database, we comprehensively evaluated glycolysis-related modification patterns in 562 DLBCL samples and analyzed their associations with immune cell infiltration and patient prognosis in the tumor microenvironment (TME). In vivo, we identified three immune subtypes in DLBCL mouse models and accessed their correlation with glycolytsis-related patterns and outcomes using histopathological evaluation (H&E) staining, Kaplan–Meier survival analysis, Western blotting, and immunohistochemistry. **Results:** Four distinct glycolysis modification patterns were identified, which correlated with three immune subtypes: immune-inflamed, immune-desert, and immune-excluded. The immune-inflamed subtype, characterized by prominent immune infiltration, was associated with favorable survival. In contrast, the immune-excluded subtype, defined by stromal activation, did not confer a survival advantage. The immune-desert subtype, marked by a lack of immune infiltration, was linked to poor survival outcomes. Finally, using the DLBCL mouse model, we validated these three immune subtypes and demonstrated their corresponding associations with glycolytic patterns and prognosis. **Conclusions:** Our work demonstrated that glycolysis-related modification patterns have correlations with the immune cell infiltration within the TME of DLBCL and that distinct glycolytic states are associated with different clinical outcomes. These findings provide a framework for improved prognostic stratification and may inform new therapeutic decision-making in DLBCL.

## Simple Summary

Diffuse large B-cell lymphoma (DLBCL) is an aggressive cancer with profound heterogeneity. While standard immunochemotherapy is curative for the majority, treatment failure in 30–40% of patients due to refractory or relapsed disease remains a significant clinical challenge, emphasizing the need for better prognostic markers. We investigated the interplay between tumor glycolytic metabolism and the tumor immune microenvironment. We identified four distinct glycolysis-related patterns that were associated with three immune subtypes (known as immune-inflamed, immune-excluded, and immune-desert). Importantly, these patterns stratified clinical outcomes and predicted chemotherapy sensitivity. For example, tumors with an immune-inflamed subtype were more sensitive to doxorubicin. Collectively, our findings propose a metabolism- and immune-based classification framework for DLBCL that may facilitate more precise risk stratification and treatment optimization.

## 1. Introduction

Diffuse large B-cell lymphoma (DLBCL) is a heterogeneous group of B-cell lymphomas characterized by substantial heterogeneity in clinical manifestations, histological morphology, and prognosis. As the most prevalent B-cell non-Hodgkin lymphoma (NHL) globally [1,2], DLBCL accounts for over 30% of all NHLs [3], with more than 150,000 new cases annually worldwide [4,5]. The high incidence and frequent recurrence of DLBCL pose significant challenges to the diagnosis and treatment [6,7,8,9,10,11].

Immunotherapy has become a significant therapy with the success of advanced molecular diagnostic platforms and the growing understanding of immune mechanisms. It has generated considerable benefits for treating a wide range of cancer types [12]. Nevertheless, R-CHOP therapy (rituximab, cyclophosphamide, doxorubicin, vincristine, and prednisone) could only achieve remission in 60% of patients during the first-line treatment of DLBCL [4,13,14], with approximately one-third of patients experiencing relapse. Variability in cancer immunotherapy responses has been attributed to factors like immune competency, diversity, different antigen specificity, and so on [15,16,17]. The complex tumor immune microenvironment (TME) plays an essential role in determining the effectiveness of immunotherapy [18,19,20,21], but the relationship between TME composition and the clinical prognosis in DLBCL is currently not fully elucidated.

In the 1920s, German scientist Otto Warburg observed that cancer cells utilize the glycolytic pathway to produce ATP even in the presence of oxygen, a phenomenon termed the “Warburg effect” [22,23,24,25,26]. In recent years, it has been demonstrated that metabolic changes occur in all cells, including those within tumor tissue, a phenomenon known as “metabolic reprogramming.” Both the Warburg effect and metabolic reprogramming highlight the importance of glycolysis in the development of cancer because glycolysis not only influences the growth and metabolism of cancer cells but also modulates the growth and infiltration of surrounding normal cells, including various stromal cells and immune cells. Therefore, an in-depth understanding of the relationship between glycolysis and DLBCL will provide insight into the diagnosis and prognosis assessment of patients with the disease.

The paper comprehensively integrated data from 562 DLBCL samples to evaluate the glycolysis modification patterns correlated with the TME cell-infiltrating characteristics. Four well-defined glycolysis modification patterns were distinguished by unsupervised clustering analysis and consensus clustering algorithm and named Gly-cluster A–D. In addition, the TME characteristics, including immune-excluded, immune-desert, and immune-inflamed subtypes, were revealed by ssGSEA and showed high consistency with the aforementioned glycolysis modification patterns above. The immune-excluded subtype was defined by matrix activation and did not associate with a better prognosis despite immune cell infiltration. The immune-desert subtype was characterized by a lack of immune infiltration, resulting in poor survival. In the DLBCL mouse model, we initially identified three immune subtypes in the DLBCL TME by H&E staining and subsequent survival analysis. Subsequently, we validated these immune subtypes and found a significant correlation between the immune subtypes and the glycolysis patterns by immunohistochemistry (IHC). Furthermore, we extracted primary cells from DLBCL tissues of mouse tissues and used them to confirm the three immune subtypes by Western blot (WB), and found that different immune subtypes have different responsiveness to doxorubicin (DOX), which correlated with different prognoses.

In summary, our work demonstrates that distinct glycolytic modification patterns in DLBCL shape the immune contexture of the TME, thereby directly influencing patient prognosis. This integrative analysis of metabolism and immunity provides a robust framework for refining prognostic assessment and guiding the development of novel combination therapies.

## 2. Methods

### 2.1. DLBCL Dataset Source and Preprocessing

All patient data and glycolysis-related gene expression profiles were downloaded from public databases. A total of 562 samples were collected from the three public datasets: CTSP-DLBCL1, NCICCR-DLBCL, and TCGA-DLBC. The comprehensive analysis, including molecular clustering, was performed on these 562 DLBCL patients. However, for survival analyses, only 191 patients with complete clinical information were included. All these samples were collected before the initiation of standard treatment following diagnosis. To ensure robust integration across the three cohorts and mitigate technical biases, all expression data were first log_2_-transformed and quantile normalized. Subsequently, the ComBat algorithm from the R package SVA (version 3.58.0) was applied to the expression matrix of the 72 glycolysis-related genes for batch effect removal, and Principal Component Analysis (PCA) was used to confirm the elimination of batch-specific clustering before downstream analyses. Clinical details, including age, gender, risk (International Prognostic Index, IPI), and prognosis, were also collected and listed in the Supplementary Files. In addition, a set of 72 glycolytic-related genes were obtained from the intersection of four glycolytic gene sets (WP, KEGG, Reactome, and Hallmark) in the MSigDB database (https://www.gsea-msigdb.org/gsea/msigdb, accessed on 20 May 2025). Ultimately, 30 genes exhibiting substantial variance were finally obtained through Median Absolute Deviation (MAD) value screening (Table 1).

### 2.2. Unsupervised Clustering for 30 Glycolysis-Related Genes

Glycolysis-related genes were sourced as described above. Based on the expression profile of the 30 selected glycolysis-related genes, unsupervised clustering analysis was performed to identify distinct glycolytic modification patterns. The consensus clustering algorithm was employed to determine the optimal number of clusters and assess their stability. The ConsensusClusterPlus package (version 1.74.0) was used to repeat the clustering process 1000 times to guarantee the robustness of the classification. Finally, four distinct glycolytic modification patterns were determined based on the expression of these 30 genes. The empirical Bayesian approach of the limma R package (version 3.66.0) was used to identify differentially expressed genes (DEGs) between the different modification patterns. A *p*-value threshold of <0.001 was set as the criterion for statistical significance. To quantify glycolytic activity at the sample level, we computed a glycolysis score (gly_score) using the glycolysis-related gene set from Table 1. For each sample, gene expression values were z-score normalized across samples, and the gly_score was defined as the mean z-score of the glycolysis genes. A higher gly_score indicates stronger glycolytic activity.

### 2.3. Gene Set Variation Analysis (GSVA) and Corresponding Functional Annotation

To explore the differences in the biological processes among these four glycolytic modification patterns, the GSVA R package (version 2.4.1) was used to perform Gene Set Variation Analysis (GSVA) enrichment. GSVA, an unsupervised and non-parametric method, is usually applied to calculate changes in biological process activities and pathways in expression data set samples. We downloaded the “c2.cp.kegg.v6.2.-symbols” gene sets from the MSigDB database to conduct the GSVA. For multi-pathway testing in GSVA, *p* values were adjusted using the Benjamini–Hochberg method, and FDR (*q*-value) < 0.05 was considered statistically significant. Subsequently, the ClusterProfiler R package was employed to perform functional annotation for glycolysis-related genes. Enrichment results were reported with FDR-adjusted *q*-values, and *q* < 0.05 was used as the significance cutoff.

### 2.4. Evaluation of the Tumor Microenvironment Cell Infiltration

The single-sample Gene Set Enrichment Analysis (ssGSEA) algorithm (version 2.0) was used to assess the relative abundance of single-cell infiltration in the TME of DLBCL. The Charoentong gene set, a predefined collection of gene signatures established by Charoentong and colleagues in 2017 [27], was applied to store different human immune cell subtypes, such as natural killer (NK) cells, T cells, macrophages, activated CD8+ T cells, regulatory T cells, and activated dendritic cells. The relevant gene signatures were obtained from this resource for marking the different TME-infiltrating immune cell types. Subsequently, ssGSEA analysis was performed to compute the enrichment score, which indicates the relative abundance of each TME-infiltrating immune cell population in the sample. To compare our glycolysis-based clusters with published DLBCL microenvironmental signatures, we computed ssGSEA scores for the functional gene expression signatures (FGES) reported by Kotlov et al. [28]. and compared scores across Gly clusters using Kruskal–Wallis tests with Benjamini–Hochberg FDR correction (*q* < 0.05).

### 2.5. The Relationship Between the Glycolysis Modification Patterns and Related Biological Processes

A series of gene sets encompassing various biological processes, including CD8+ T-effector signature, immune checkpoint, angiogenesis signature, WNT targets, and mismatch repair, was utilized. Subsequently, a correlation analysis was performed to further elucidate the relationship between the glycolysis gene characteristics and these related biological pathways.

### 2.6. In Vivo Experiments in Mice with DLBCL

#### 2.6.1. DLBCL Mouse Model

Female NOD mice (20–24 weeks of age) were purchased from Nanjing Qinglongshan Animal Breeding Farm (Nanjing, China). The ethical review number of this experiment was 2020~Gl18. Mice were subcutaneously injected with 0.1 mL of cell suspension containing 10^7^ SUDHL-4 cells on one side of the rib cage. (*n* = 24). After injection, the mice were monitored daily, and their body weights were recorded. 10 days later, tumor-bearing mice were separated from non-tumor-bearing mice, and doxorubicin (DOX) 0.5 mg/kg was administered intraperitoneally to the DLBCL model mice (*n* = 20). The treatment regimen involved three consecutive daily doses followed by a one-day intermission, repeated for three weeks. Body weights were recorded throughout the treatment period.

#### 2.6.2. Differentiation of Immune Subtypes

DLBCL mouse tissues were collected, and the DLBCL model mice were classified into immune-desert, immune-excluded, and immune-inflamed groups based on H&E staining and survival analysis. The immune-desert group was defined as a “barren” area characterized by scant cell infiltration, few spindle-shaped fibroblasts, loose mature fibers, and often presenting with keloid or mucinous features; the immune-inflamed group was defined as a “reactive” area containing abundant fibroblasts with enlarged nuclei, minimal non-cellular components, and typically enriched with inflammatory infiltrates; the immune-excluded group was defined as an intermediate subtype between the two above-mentioned groups.

#### 2.6.3. Analysis of the Immune Subtypes

The immune subtypes identified by H&E staining and survival analysis were verified by measuring the expression level of anti-mouse CD3, CD20, CD68, α-SMA, and CD31 (Biotime, Shanghai, China) via immunohistochemistry (IHC). Specifically, the immune-desert group exhibited high CD20 expression, while the immune-inflamed group showed high expression levels of CD3, CD68, α-SMA, and CD31.

#### 2.6.4. Glycolysis Pattern Analysis

Glycolysis patterns were validated and characterized by IHC. According to the results of bioinformatics analysis, Gly-clusters A and B exhibited higher levels of ENO2 expression compared to the other groups; Gly-cluster C exhibited significantly higher levels of PFKM expression than the remaining groups; and Gly-cluster D exhibited higher levels of NUP35 than the other groups.

### 2.7. Primary Cell Experiments

#### 2.7.1. DLBCL Primary Cell Extraction and Culture

The obtained tumor tissue was carefully dissected to remove fat, connective tissue, and necrotic parts. It was washed three times in a flat dish with pre-cooled sterile Phosphate-Buffered Saline (PBS) (Biotime, Shanghai, China), and subsequently placed in a pre-cooled culture medium for transport to the laboratory. The tissue was trimmed and cut into 1–2 mm^3^ small pieces. Inoculate in culture flasks (or dishes) and incubate at 37 °C, 5% CO_2_, or cover and plug in a normal thermostat. The cells were cultured in Roswell Park Memorial Institute (RPMI) 1640 medium (Gibco, New York, USA) supplemented with 10% Fetal Bovine Serum (FBS) (Gibco, New York, USA), penicillin, and streptomycin at a final concentration of 10 U/mL.

#### 2.7.2. Cellular Experiments

Immune subtypes were confirmed by Western blot (WB), which measured the levels of CD3, CD20, CD68, α-SMA, and CD31 (Biotime, Shanghai, China).

For the cell viability assay, primary DLBCL cells were inoculated in 96-well plates at a cell density of 1 × 10^4^ cells per well. After 48 h in the induction medium, the medium was discarded. Subsequently, the cells were treated with DOX at varying concentrations (1 ng/mL, 2.5 ng/mL, 5 ng/mL, 10 ng/mL, 20 ng/mL, 50 ng/mL, or 100 ng/mL) and incubated for a total of 4 h. Cell viability was quantified using a standard CCK-8 assay (Beyotime, Shanghai, China), and the data represent the average of three parallel measurements.

To measure apoptosis, the DLBCL primary cells were treated with 100 ng/mL DOX for 4 h, and then were stained with an Annexin V-FITC/PI Apoptosis Kit (Abbkine, Wuhan, China). Flow cytometry (FCM) determined the degree of apoptosis, and the cells were defined as early and late apoptotic cells.

### 2.8. Statistical Analysis

Distance correlation and Spearman correlation analysis were used to assess the association between the expression of glycolysis regulators and TME-infiltrating immune cells. In addition to the One-way Analysis of Variance (ANOVA,using the IBM SPSS Statistics software Version 29.0), the Kruskal–Wallis test was also applied to conduct difference tests between these groups. The Kaplan–Meier method was used to generate survival curves for predictive analysis, and the log-rank test was applied to identify the significance of the differences. For analyses involving multiple hypotheses (e.g., pathway/signature correlations and immune-signature comparisons), *p* values were adjusted using the Benjamini–Hochberg method to control FDR, and *q* < 0.05 was considered significant unless otherwise specified. All statistical *p* values were two-sided; *p* < 0.05 was considered statistically significant. R statistical software (version 3.6.1) was used to process all the data.

## 3. Results

### 3.1. Identification of Four Prognosis-Associated Glycolysis Modification Patterns via Unsupervised Clustering in DLBCL

**Figure 1:**
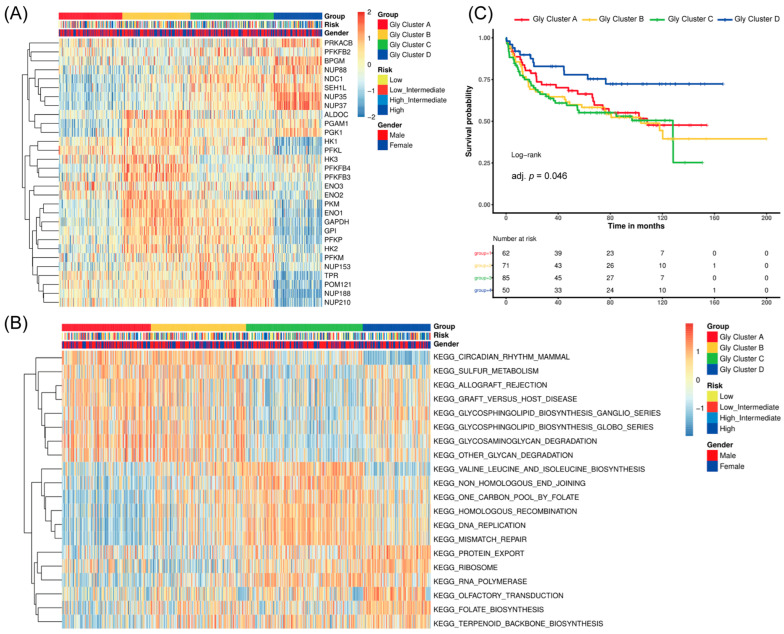
Cluster analysis of glycolysis. (**A**) Unsupervised clustering of 30 glycolysis-related regulators in the DLBCL cohort. The gender, Gly cluster, and risk were used as patient annotations. High expression is marked in yellow, while low expression in blue. (**B**) Unsupervised clustering of glycolysis-related signaling pathway in the DLBCL cohort. The cluster, risk, and gender were used as patient annotations. High expression is marked as yellow, while low expression as blue. (**C**) Kaplan–Meier survival analysis of the four Gly modification patterns. While the unsupervised clustering was performed on the full cohort (*n* = 562), the survival analysis was restricted to the subset of patients with available follow-up data (*n* = 191). The Kaplan–Meier analysis showed a significant difference in overall survival among the four Gly modification patterns (log-rank *p* = 0.011). Gly cluster-D showed significantly better overall survival than the other three clusters.

72 glycolysis-related genes were obtained from the intersection of four glycolysis gene sets in the MSigDB database. Through MAD value screening, 30 genes with significant differences were finally selected for the following research (Table 1). The patients with different glycolysis modification patterns were distinguished using the R package ConsensusClusterPlus and ranked based on the 30 glycolysis-related genes. Four different patterns were finally determined through unsupervised clustering, including 131 cases in type A, 151 cases in type B, 164 cases in type C, and 118 cases in type D, respectively. Evidently, in glycolysis modification pattern A (Gly cluster A), the expression of glycolysis-related genes like PFKL, HK3, and ENO2 were relatively high. In Gly cluster B, in addition to the above genes, the expression level of ALDOC, PGAM1, and PFKFB4 was also increased. This result indicates there may be some similarities between Gly cluster A and Gly cluster B. While in Gly cluster C, the expression level of PFKM, TPR, POM121, and NUP188 was higher than in other clusters. Cluster D was significantly different from the other three groups because the expression of genes mentioned above was all decreased. In contrast, the expression of genes, including PRKACB, BPGM, NUP35, and NUP37, was higher than the other three clusters (Figure 1A). In another way, the signaling pathway cluster analysis of these genes also showed significant differences between Gly cluster A, Gly cluster B, and the other two groups (Figure 1B), which strengthened our hypothesis about Gly cluster A and B. Results above confirmed the existence of glycolysis modification patterns in DLBCL. Moreover, the subsequent survival probability of these four clusters showed that cluster D had a better survival advantage than the other three clusters (Figure 1C). The Supplementary Tables S1 and S2 reported Cox HRs/95%CIs; Gly-D showed improved OS vs. Gly-A (HR 0.257, 95% CI 0.0856–0.768), while non-significant D vs. B/C contrasts had wide CIs likely due to limited events. The reason was discussed in the following research.

### 3.2. Glycolysis Modification Patterns Drive Heterogeneous Immune Cell Infiltration in the TME

**Figure 2:**
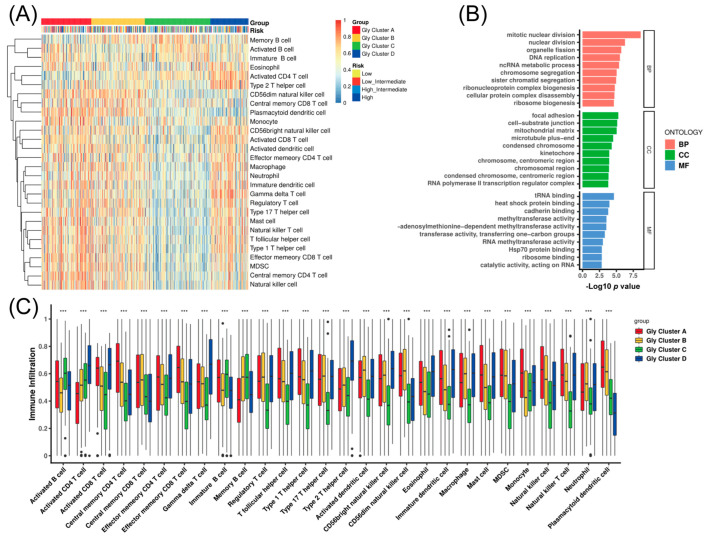
Immune infiltration analysis. (**A**) Unsupervised clustering of multiple immune cell types across the four Gly clusters. Cluster membership and risk group were used as patient annotations. High expression is shown as yellow, whereas low expression is shown in blue. (**B**) Gene Ontology (GO) enrichment analysis of Gly genes to characterize functional annotation. Bar length indicates the number of enriched genes. (**C**) Abundances of tumor TME–infiltrating cells across the four Gly modification patterns. In the boxplots, the upper and lower bounds denote the interquartile range, the center line indicates the median, and black dots represent outliers. Asterisks indicate statistical significance ( *** *p* < 0.001). BP, biological process; CC, cell component; MF, molecular function.

Glycolysis produces a large amount of lactic acid that can acidify the TME, thereby influencing immune cells’ distribution, so we next explored the TME cell infiltration features in different glycolysis modification modes. To achieve this, immune cell signatures curated by Charoentong et al. [27] were applied to store immune cell subtypes, and the ssGSEA algorithm was applied to estimate the relative abundance of single-cell infiltration and obtain enrichment scores. Subsequent analyses of TME cell infiltration revealed that Gly cluster A was enriched for innate immune cell infiltration, including mast cells, natural killer (NK) cells, Myeloid-derived suppressor cells (MDSCs), and macrophages (Figure 2A). However, patients in this cluster did not exhibit improved survival (Figure 1C). Consistent with prior reports, immune-excluded tumors may still display substantial immune infiltration [29]. In this context, immune cells are predominantly retained within the peritumoral stroma, with limited access to the tumor parenchyma. Accordingly, stroma activation has been proposed to impede effective T-cell penetration, although the precise contribution of stromal components to immune suppression in the TME remains to be fully elucidated. Similarly, Gly cluster B showed increased infiltration of innate immune cells (e.g., MDSCs and NK cells) yet was not associated with a survival advantage. This further supports our earlier hypothesis: Gly cluster C, characterized by low immune infiltration, was associated with the poorest survival. By contrast, Gly cluster D showed increased infiltration of activated CD4 T cells, effector memory CD4 T cells, and activated CD8 T cells, consistent with a more favorable prognosis (Figure 2B). Collectively, these results indicate that the four glycolysis expression patterns are accompanied by distinct TME immune landscapes. Accordingly, Gly clusters A and B were grouped as an immune-excluded subtype, featuring predominant innate immune infiltration alongside stromal activation; Gly cluster C was defined as an immune-desert subtype, with suppressed antitumor immunity; and Gly cluster D was categorized as an immune-inflamed subtype, characterized by effective immune-cell infiltration and immune activation. We further scored Kotlov et al. FGES by ssGSEA and observed concordant patterns across Gly clusters (Supplementary Figure S1): Gly-A/B were enriched for stromal/ECM programs (immune-excluded/stroma-activated), Gly-C showed low immune signals (immune-desert), and Gly-D showed immune-activation/trafficking (immune-inflamed). We therefore use these descriptive aliases for clarity without changing sample membership.

Moreover, to explore the biological programs underlying the glycolysis modification patterns, we applied the ClusterProfiler package to perform the GO enrichment analysis. The results demonstrated that multiple biological processes were significantly associated with the glycolysis-related clusters, providing additional evidence that these modification patterns are closely linked to immune regulation within the TME (Figure 2C).

### 3.3. Cross-Database Validation Confirms Reproducible Glycolysis Patterns in DLBCL

**Figure 3:**
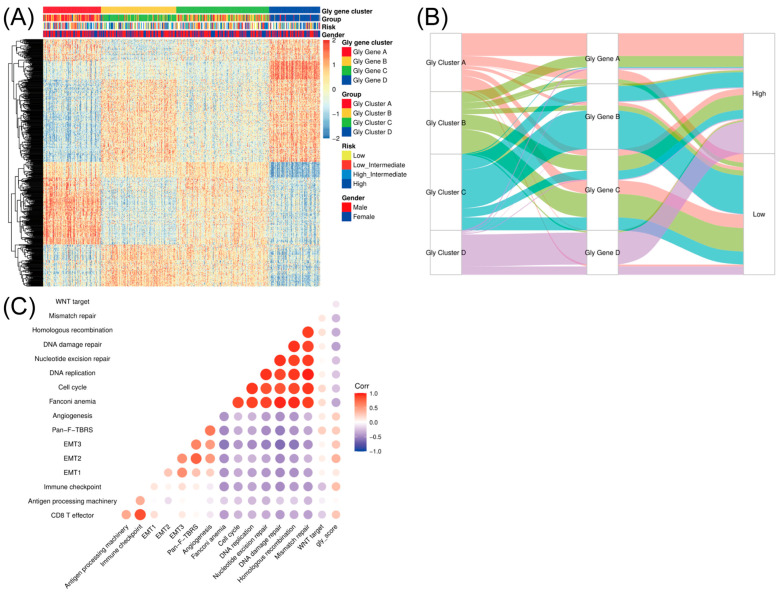
Database validation. (**A**) Unsupervised clustering of glycolysis-related genes in the merged DLBCL cohort to classify patients into different genomic subtypes, termed as Gly gene cluster **A**–**D**, respectively. (**B**) Using the alluvial diagram to show the changes in Gly clusters, Gly gene clusters, and disease risks. (**C**) Using Spearman correlation analysis to show correlations between glycolysis and some known gene signatures in the merged DLBCL cohort. Negative correlations were marked with blue and positive correlations with orange.

To further validate these glycolysis-related modification patterns, we performed unsupervised clustering based on the glycolysis-related gene set in the merged DLBCL cohort (CTSP-DLBCL1, NCICCR-DLBCL, and TCGA-DLBC, after batch correction as described in the Methods) to classify patients into different genomic subtypes. The results were very much in line with our expectations. Consistent with the glycolysis modification patterns identified above, the unsupervised clustering algorithm revealed four reproducible glycolysis genomic subtypes, which we termed Gly gene clusters A–D (Figure 3A), supporting the existence of four glycolysis-related transcriptional programs in DLBCL. It was observed that among the four groups of glycolysis gene clusters, Gly gene clusters A and C and Gly gene clusters B and D had significant differences. This phenomenon is entirely consistent with our modification clustering in glycolysis because Gly clusters A and B exactly correspond to Gly gene clusters A and C. The alluvial diagram used to visualize the attribute changes in individual patients was also proof of this result (Figure 3B). Furthermore, the correlation between the known signatures and the glycolysis modification was also tested to better illustrate the characteristics of glycolysis-related modification patterns (Figure 3C). Spearman correlation analysis showed significant correlations between glycolysis-related subtypes and multiple known signatures, further supporting the biological relevance of our glycolysis-driven classification.

### 3.4. Experimental Validation Links Glycolysis Patterns to Immune Subtypes and Chemotherapy Response

**Figure 4:**
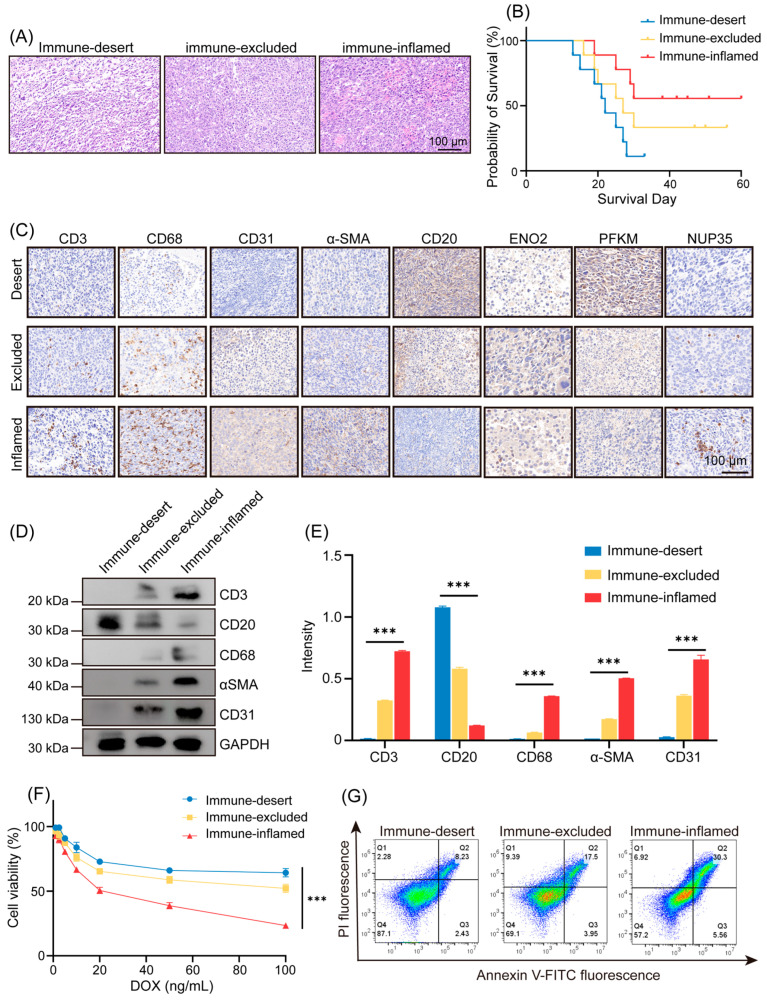
Identify three immune subtypes in DLBCL tissues, and confirm their correlation with glycolysis patterns and prognosis. (**A**) H&E staining to identify three immune subtypes. (**B**) Survival analysis to identify three immune subtypes. (**C**) IHC test to analyze the expression level of CD3, CD68, CD31, α-SMA, and CD20, which are related to immune subtypes identification; and ENO2, PFKM, and NUP35, which are related to different glycolysis patterns. All IHC markers in Figure 4C were detected using mouse-reactive antibodies to evaluate host TME components in the xenograft tumors. (**D**) In vitro, detection of protein expression by WB (The unedited original images can be found in Supplementary Material File S1). (**E**) Quantitative analysis of the WB assay, *** *p* < 0.001. (**F**) CCK-8 determination of the responsiveness of different immune subtype cells to stepped concentrations of DOX. (**G**) Apoptosis of different immune subtype cells was determined by flow cytometry after treatment with 100 ng/mL DOX for 4 h.

To verify the bioinformatic analysis results above, we established a DLBCL xenograft mouse model and conducted in vivo experiments. Based on H&E staining and survival analysis, we identified three immune subtypes and categorized the DLBCL mice accordingly (Figure 4A,B). The immune-desert group was characterized by minimal inflammatory infiltration and limited fibroblast involvement and was associated with poor survival. In contrast, the immune-inflamed group exhibited prominent immune infiltration accompanied by an activated stromal component and showed more favorable prognosis. The immune-excluded group displayed an intermediate pattern, characterized by immune/stromal features between the above two phenotypes. Two mice were excluded due to early mortality within the first three days following DOX administration. The final distribution of mice corresponding to the immune subtypes was: immune-desert (*n* = 8), immune-excluded (*n* = 6), and immune-inflamed (*n* = 4). After identifying three immune subtypes, the IHC was used to confirm the identification. Results showed that the immune-desert group exhibited higher level of CD20, while the immune-inflamed group showed increased CD3, CD68, CD31, α-SMA, which was consistent with bioinformatics analysis and in the studies (Figure 4C). To further explore the relationship between immune subtypes and glycolysis patterns, we assessed the IHC expression of representative signature proteins associated with each glycolysis pattern, including ENO2, PFKM, and NUP35. ENO2, a marker enriched in Gly clusters A and B, was predominantly expressed in the immune-excluded subtype; PFKM, a marker of Gly cluster C, was highly expressed in the immune-desert subtype; and NUP35, a marker for Gly cluster D, was highly expressed in the immune-inflamed subtype (Figure 4C). Overall, these findings support the concordance between the mouse-model phenotypes and our bioinformatic classification.

In vitro, we isolated tumor cells from tumor-bearing mice to test the protein expression levels by Western blotting, which was consistent with the IHC findings (Figure 4D,E). In addition, we tested the DOX sensitivity across three different immune subtypes. Dose–response assays showed that cells from the immune-inflamed group were the most sensitive to DOX, exhibiting a 2.36-fold higher response than cells from the immune-desert group (Figure 4F). Consistently, flow cytometry demonstrated that DOX induced 3.68-fold higher late apoptosis and 2.29-fold higher early apoptosis in the immune-inflamed group than in the immune-desert group (Figure 4G).

In summary, our in vivo and in vitro results support the presence of three distinct immune phenotypes in DLBCL xenografts. These phenotypes are associated with differential glycolysis-related protein signatures (ENO2, PFKM, and NUP35) and exhibit marked differences in survival outcomes and chemotherapy sensitivity.

## 4. Discussion

The Warburg effect in tumor cells has been proven to influence the tumor microenvironment significantly [22,23,24,25,26]. Therefore, it is of great significance to further study glycolytic reprogramming in tumor tissues for improving diagnosis and prognosis. Here, based on 30 glycolytic genes, we identified four different glycolytic modification patterns. Among them, cluster D showed the best survival advantage, while cluster C showed the worst survival. Subsequently, analysis of immune cell infiltrates in each cluster showed that these four glycolytic modification patterns correspond to three different characteristics of TME cell infiltration. In these four patterns, cluster A and B were characterized by innate immune infiltration and stromal activation, corresponding to the immune-excluded subtype; cluster C showed suppressed immune activity, corresponding to the immune-desert subtype; cluster D exhibited adaptive immune activation, which was described as the hot tumor, corresponding to the immune-inflamed subtype. Furthermore, we used additional datasets to validate the robustness of this glycolysis pattern. Results from other databases showed that there were also four groups in the classification model and that the grouping and prognosis of each group were similar to that of the glycolysis cluster group. To further corroborate our findings, we performed in vivo and in vitro experiments and identified three immune subtypes in the DLBCL mice model. Through in vivo experiments, we confirmed the concordance between immune subtypes and glycolysis patterns. In vitro, we demonstrated that cells from different immune subtypes displayed distinct responsiveness to doxorubicin (DOX).

In addition, we noted that there had been studies that classified immune infiltration subtypes in the immune microenvironment of DLBCL [28], and their results are similar. In the article mentioned above, DLBCL was classified into two subtypes, GCB-DLBCL and ABC-DLBCL, and four immune cell-infiltrating subtypes were analyzed, namely, germinal center-like (GS), mesenchymal (MS), inflammatory (IN), and depleted (DP). Their GS cluster corresponded to our immune-inflamed cluster, which showed abundant immune cell infiltration and favorable survival. In contrast, the DP cluster corresponded to our immune-desert cluster, which showed low immune cell infiltration and poor prognosis. The other two categories also corresponded to our immune-excluded cluster, i.e., characterized by stromal activation and lacking a survival benefit. The results above further support the robustness of our results.

Notably, the literature on immune/stromal infiltrates in DLBCL is extensive and sometimes yields heterogeneous conclusions depending on cohort composition, methodology, and therapeutic context. Shipp et al. established, using gene-expression profiling, that DLBCL is biologically heterogeneous and can be stratified into reproducible molecular risk groups beyond conventional clinicopathologic variables [30]. Lenz et al. further showed that the non-malignant microenvironment contributes independent prognostic information, with distinct stromal programs associated with divergent outcomes [31]. Riihijärvi et al. highlighted that the prognostic impact of specific immune infiltrates such as CD68+ macrophages may be treatment-dependent [32]. Perry et al. translated microenvironment biology into an IHC-based prognostic model using stromal surrogates and microvascular density [33]. Staiger et al. developed an FFPE-compatible microenvironment-focused expression signature that stratifies risk independently of established factors [34]. Collectively, these studies frame the DLBCL TME as context-dependent and motivate our glycolysis-driven immune subtypes as a complementary layer linking metabolic states to distinct immune landscapes.

Furthermore, for the glycolysis-driven TME classification to be translated into clinical practice as a prognostic factor, it needs to be compared with existing clinical schemes, such as the International Prognostic Index (IPI) and Cell-of-Origin subtype (COO) subtype, to achieve more refined risk stratification. As this study relies on bioinformatic analysis and small-sample murine experiments, it lacks extensive clinical cohorts and a simple scoring algorithm, which consequently limits its immediate clinical implementation. Future clinical validation studies must prioritize developing a minimal gene panel and a standardized scoring algorithm for practical use (e.g., via qPCR), and systematically comparing the combined prognostic power of our glycolysis model with existing tools (IPI, COO) to demonstrate its added clinical value.

## 5. Conclusions

In conclusion, this study innovatively applies glycolytic clustering to classify DLBCL patients, revealing distinct patterns of immune infiltration that are strongly linked to clinical outcomes. These findings, validated through in vitro and in vivo models, lay the groundwork for refining diagnosis, subtyping, and prognosis in DLBCL patients.

## Figures and Tables

**Table 1:** Thirty glycolysis-related genes were selected from the intersection of four glycolytic gene sets (WP, KEGG, Reactome, and Hallmark) in the MSIGDB database. These genes were further filtered for substantial variance using the MAD value screening.

Selected Glycolysis-Related Genes (n = 30)				
HK1	NUP35	GPI	PFKP	SEH1L
HK2	NUP37	TPR	PFKM	GAPDH
HK3	NUP88	PKM	PFKL	ALDOC
ENO1	NUP153	PFKFB2	PGK1	PGAM1
ENO2	NUP188	PFKFB3	BPGM	PRKACB
ENO3	NUP210	PFKFB4	NDC1	POM121

## Data Availability

All the data generated and analyzed during this study are included in the manuscript.

## Supplementary Materials

The following supporting information can be downloaded at: https://www.mdpi.com/article/10.3390/cancers18010075/s1, Figure S1: Kotlov FGES ssGSEA scores compared across Gly clusters (Kruskal–Wallis; BH-FDR q-values). Heatmap shows row z-scored mean ssGSEA scores per cluster; higher values indicate relative enrichment of the corresponding microenvironmental program; Table S1. Univariate Cox regression for overall survival across glycolysis clusters (reference = Gly-A); Table S2. Pairwise Cox regression comparisons of Gly-D versus other glycolysis clusters; File S1: Unedited original Western Blot images.
