# Supplementary material for: Glycolysis-Driven Immune Subtypes in the Tumor Microenvironment Determine Clinical Outcomes in Diffuse Large B-Cell Lymphoma
Source: Cancers (Basel). 2025 Dec 25;18(1):75. doi: 10.3390/cancers18010075 (PMC12784748; doi:10.3390/cancers18010075)

Supplementary Table S1. Univariate Cox regression for overall survival across glycolysis clusters (reference = Gly-A).

model	estimate	conf.low	conf.high	p.value
Gly-B	0.6853	0.0867	5.4183	0.7202
Gly-C	0.4004	0.1484	1.0804	0.0707
Gly-D	0.2565	0.0856	0.7685	0.0151

Supplementary Table S2. Pairwise Cox regression comparisons of Gly-D versus other glycolysis clusters.

comparison	estimate	conf.low	conf.high	p.adj
Gly-D vs Gly A	0.2622	0.0877	0.7842	0.0461
Gly-D vs Gly B	0.3267	0.0376	2.8399	0.3106
Gly-D vs Gly C	0.6444	0.2045	2.0405	0.4530

Supplementary Figure S1. Kotlov FGES ssGSEA scores compared across Gly clusters (Kruskal–Wallis; BH-FDR q-values). Heatmap shows row z-scored mean ssGSEA scores per cluster; higher values indicate relative enrichment of the corresponding microenvironmental program.

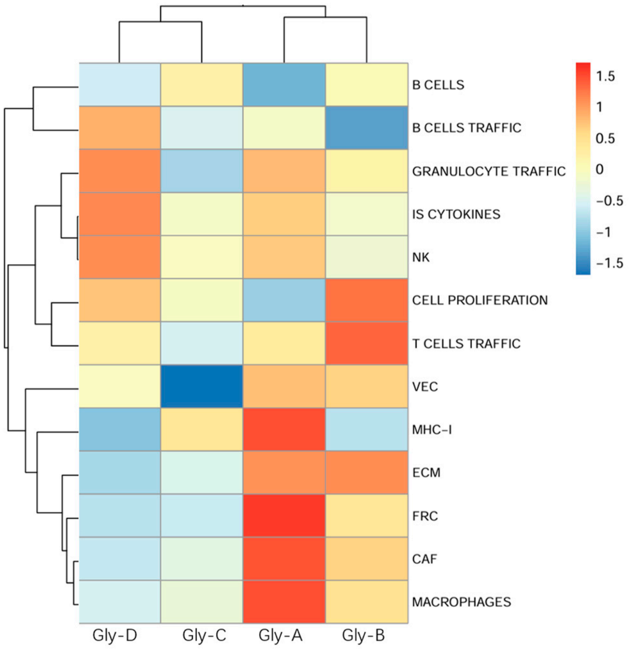

Supplement: Supplementary file 1 [file cancers-18-00075-s001.zip › cancers-4015961-Supplementary Tables and Figure.pdf]
