# Supplementary material for: Glycolysis-Driven Immune Subtypes in the Tumor Microenvironment Determine Clinical Outcomes in Diffuse Large B-Cell Lymphoma
Source: Cancers (Basel). 2025 Dec 25;18(1):75. doi: 10.3390/cancers18010075 (PMC12784748; doi:10.3390/cancers18010075)

Dear editor,

Thank you very much for your patient review and constructive suggestions. We appreciate your attention to detail regarding the Western Blot data presentation.

The images below present our Western Blot results, including the corresponding densitometry readings and intensity ratios for each band.

We must note that the full membrane image for the CD31 blot is unavailable. Given the high molecular weight (130-140 kDa) of the target protein, a wide-range protein ladder and maximal electrophoretic separation were necessary. To achieve optimal band resolution, the lower portion of the membrane, which contained the irrelevant low molecular weight markers, was excised prior to imaging. We have, however, ensured the complete integrity of the upper part of the membrane, which sufficiently encompasses the region of interest and verifies the molecular weight of CD31.

For full context, we have included the complete, uncropped blots (whole blots) alongside molecular weight markers for all experiments in the Supplemental Materials section.

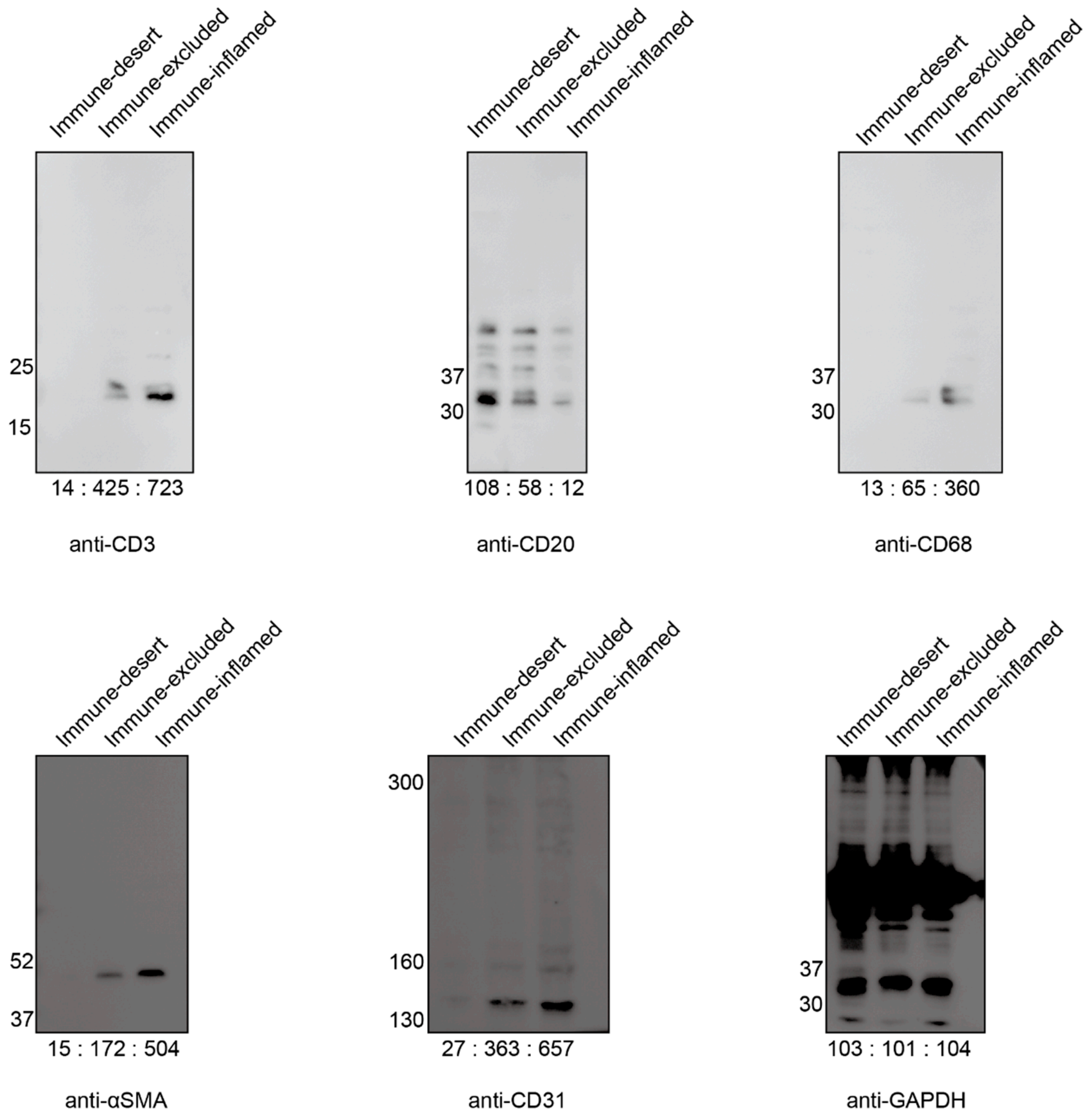

Supplement: Supplementary file 1 [file cancers-4015961-File S1-Unedited original Western Blot images .pdf › cancers-4015961-File S1-Unedited original Western Blot images .pdf]
